# A ‘major breakthrough’, yet potentially ‘entirely ineffective’? Experts’ opinions about the ‘total ban’ on unhealthy food marketing online in the UK’s Health and Care Act (2022)

**DOI:** 10.1017/S0007114523002829

**Published:** 2025-03-28

**Authors:** Jennifer L. Harris, Emma Boyland, Magdalena Muc, Louisa Ells, Jayne Rodgers, Zoe Hill, Victoria Targett, Michelle Young, Mimi Tatlow-Golden

**Affiliations:** 1 Rudd Center for Food Policy & Health, University of Connecticut, Hartford, CT, USA; 2 Department of Psychology, University of Liverpool, Liverpool, L69 7ZA, UK; 3 Faculty of Wellbeing, Education and Language Studies, The Open University, Milton Keynes, MK7 6AA, UK; 4 Obesity Institute, Leeds Beckett University, Leeds, LS1 3HE, UK; 5 Teesside University International Business School, Teesside University, Middlesbrough, TS1 3BX, UK; 6 Office for Health Improvement & Disparities, Department of Health and Social Care, London, SW1H 0EU, UK

**Keywords:** Digital marketing, HFSS marketing, Government regulation, Expert interviews, Policy design

## Abstract

The UK’s Health and Care Act (2022; paused until 2025) includes a globally novel ban on paid-for online advertising of food and beverage products high in saturated fat, salt and sugar (HFSS), to address growing concerns about the scale of digital marketing and its impact in particular on children’s food and beverage preferences, purchases and consumption. This study aimed to understand the potential impact of the novel ban (as proposed in 2020) on specified forms of online HFSS advertising, through the lens of interdisciplinary expertise. We conducted semi-structured interviews via videoconference with eight purposively selected UK and global digital marketing, food and privacy experts. We identified deductive and inductive themes addressing the policy’s scope, design, implementation, monitoring and enforcement through iterative, consensual thematic analyses. Experts felt this novel ‘breakthrough’ policy has potential to substantially impact global marketing by establishing the principle of no HFSS advertising online to consumers of all ages, but they also identified substantive limitations that could potentially render it ‘entirely ineffective’, for example, the exclusion of common forms of digital marketing, especially brand marketing and marketing integrated within entertainment content; virtual/augmented reality, and ‘advertainment’ as particularly likely spaces for rapid growth of digital food marketing; and technical digital media issues that raise significant barriers to effective monitoring and compliance. Experts recommended well-defined regulations with strong enforcement mechanisms. These findings contribute insights for effective design and implementation of global initiatives to limit online HFSS food marketing, including the need for government regulations in place of voluntary industry restrictions.

Extensive marketing of food and non-alcoholic beverages (hereafter: food) high in saturated fat, salt and sugar (HFSS) contributes to poor diets in children (including adolescents)^(1–3)^ and has been strongly implicated in the development of obesity and other diet-related diseases^(4–8)^. The WHO^(2)^ has called on member states to regulate HFSS food marketing to children as a public health priority, yet few governments have implemented regulations to reduce children’s exposure to and/or the power (i.e. use of persuasive techniques) of HFSS food marketing^(9,10)^. Moreover, most existing regulations are limited in scope, primarily covering TV advertising to children under age 13 and/or marketing in schools^(10)^. Thus, children worldwide continue to be exposed to vast amounts of HFSS food marketing in the media and their communities^(3,11–13)^.

With the rapid rise of digital media, children in many regions now spend more time on mobile devices compared with traditional TV viewing^(14,15)^. Advertising has proliferated online, and companies have pioneered sophisticated and innovative techniques to promote products^(16,17)^. Many digital marketing practices raise concerns, especially when targeted to children. In addition to promoting HFSS foods and other harmful products (e.g. alcohol, tobacco, gambling), online marketing techniques often exploit children’s vulnerabilities^(16,18)^. For example, marketing is disguised as entertainment (e.g. embedded within games and videos), spreads through peer networks (e.g. social media posts) and features popular celebrities and cultural themes (e.g. music, sports) to appeal to young people’s emotions and developing identities. Digital marketing uses children’s personal data and monitors their online behaviours to target marketing messages^(19)^. These common data-driven marketing techniques threaten children’s rights to privacy as well as their dietary health^(16)^. Moreover, individuals in lower-income households (in the UK and globally) experience greater exposure to unhealthy food advertising and greater risk of obesity and related diseases^(20)^, contributing to health disparities.

Industry organisations have taken some steps regarding HFSS digital marketing to children. In 2017, the UK’s industry self-regulatory Committee of Advertising Practice (CAP) developed rules that banned advertising for HFSS products in ‘children’s media’ online^(21)^, defining this as online platforms aimed at children (e.g. YouTube Kids) or other content for which children under 16 years make up more than 25 % of the audience (e.g. a YouTube influencer video with a substantial child audience)^(22)^. In 2020, Google introduced a policy of only serving HFSS marketing on their platforms in the UK and EU to users with a declared age of over 18 years. Yet the CAP rules have not eliminated HFSS marketing to children^(22)^, and self-regulatory policies have been criticised for lack of oversight and external monitoring^(19,23)^.

The UK Government has also acted to address the HFSS advertising environment. In 2007, it introduced legally binding regulations to reduce children’s exposure to HFSS television advertising^(24)^, defining HFSS through a Nutrient Profile Model^(25)^. In July 2020, it announced consultation on a ban on all HFSS product advertising on TV and online before 9 pm (not only child-specific settings) ^(26)^, and in November 2020 it further proposed a globally novel restriction^(27)^ that would ban all paid online HFSS product (but not brand) advertising including banner and video ads and other types of marketing that companies pay for to reach all consumers. The November 2020 proposal included ‘owned’ media (i.e. the company’s websites and social media pages, which were not included in the final 2022 Act), as well as influencer marketing and some other formats (see online Supplemental Materials) and applied to advertising that features products, not brands overall, as restricted items are identified through nutrient profiling.

These initiatives were part of a new UK obesity strategy^(26)^ aimed at addressing alarming rates of obesity among adults as well as children, and including measures designed to ‘help people live healthier lives’ and resulted in the Health and Care Act (2022). However, some of the features of the ‘total’ online ban as proposed in November 2020^(27)^ were not included in the Act, as discussed further below. Note also that the commencement of the online ban has, at the time of writing, been paused until 2025.

The originally proposed ban would be novel globally, although there are several privacy-related measures in the UK and elsewhere are relevant to online advertising regulation more generally. The voluntary UK Age-Appropriate Design Code (AADC)^(29)^ calls for default privacy protections for children under age 18 on all digital platforms. Beyond the UK, the US Children’s Online Protection and Privacy Act (COPPA)^(30)^ and the EU General Data Protection Regulation (GDPR)^(31)^ currently require parental permission to collect personal data from children under age 13 (up to 16 in some EU member states). California has implemented, and other US states are considering, children’s online privacy legislation modelled on the AADC. The 2022 EU Digital Services Act (effective 2024) will ban online advertising targeted to children under 18 and restrict use of data for profiling^(32)^.

Common to all these regulations and laws are challenges inherent in designing policy to regulate digital marketing. These include specifying its scope and implementation^(9,16,33–35)^. Effective implementation (including monitoring and enforcement) is especially challenging in the digital marketing ecosystem, where advertising is sold and placed through millisecond auctions, with implications for compliance monitoring^(16)^.

The purpose of this qualitative study was to provide insight into the potential efficacy of the design and implementation of the online ban as proposed in November 2020, of paid and owned HFSS product advertising (including on social and health inequities), building on existing evidence on challenges and lack of progress in this policy area^(9,16)^. Bringing global expert insights to bear on the challenge of designing online regulation has relevance for global stakeholders seeking to address this major regulatory challenge. Therefore, this study was guided by the following questions:

1. Scope: How can the scope of the UK ‘total’ online HFSS ad ban as originally proposed in November 2020^(27)^ be best defined?
2. ‘Future-proofing’: How might online HFSS ad bans be future-proofed (i.e. designed so as to remain effective despite anticipated developments in marketing technology, delivery and strategy, and to close loopholes)?
3. Implementation: What are the barriers and potential levers to digital food marketing regulation being implemented, monitored and enforced?

## Methods

Consulting with diverse experts (political, commercial, technical and practical), we aimed to generate insights regarding the design and potential impact of the novel UK HFSS online paid advertising ban. The interviews were carried out when the policy had originally been proposed, during the consultation process^(27)^ and was discussed with the experts prior to the government consultation response^(28)^ which removed some of the originally proposed restrictions and shaped the final Health and Care Act (2022). Therefore, the proposed ban as discussed by these interviewees was stronger than the ban in the 2022 Act’s final iteration.

Expert consultation is a qualitative method for identifying key features and potential routes to progress on a given topic, typically used in fields where knowledge is uncertain^(36,37)^. This approach complements previous studies examining the challenges of research and regulation in digital marketing of HFSS food to children^(9,32)^. We conducted expert interviews using a semi-structured interview guide to provide insights into these research questions. Due to the wide geographic distribution of the experts, individual interviews were conducted via video conference.

### Identification of experts

A purposive list of global experts was developed through the research team’s knowledge of stakeholders in the field, as well as discussions with an Expert Advisory Group commissioned by Public Health England (PHE), and review with PHE. We aimed to recruit expert participants with a range of relevant expertise, including marketing, digital technology and regulatory policy (obesity, food, marketing and/or privacy). Experts were also recruited from a range of countries, ensuring inclusion of experience of food marketing policy cycles in countries in the wider European Region and beyond.

Eleven experts were contacted, and eight agreed to be interviewed. Two experts based in Canada declined as they felt that their lack of success in passing such regulation did not support commentary on the UK digital plans. One additional expert did not respond. The eight participating experts were based in the UK, North America, South America and Europe, all with global expertise in digital media and/or marketing policy (see Table 1). Their areas of expertise spanned diverse combinations of health and nutrition, HFSS marketing (creative, business development and analysis), digital marketing, surveillance-based digital advertising, digital and mobile media, consumer affairs, children’s rights, policy and regulation development and analysis. All had NGO affiliations interested in monitoring and/or regulation to protect child health and well-being in digital media. Some had previous or current industry expertise. Participants provided informed consent, including consent to be quoted. Further information about interviewees’ expertise can be found in the online Supplemental Materials.

**Table 1.** Expert interviewees by area of expertise and region

Area of expertise	Regions represented
Marketing/digital industry	UK, Global
Marketing and/or children/obesity policy	South America, North America, UK, Europe
Digital privacy policy	Europe, UK, North America

### Procedures

All eight interviews were conducted by one study author (MTG), whose expertise lies in the field of transdisciplinary digital food marketing to children and children’s rights. Ethical clearance for the interviews was granted by the University of Liverpool Health and Life Sciences Research Ethics Committee in March 2021 (reference 9869). Interviews were conducted in 2021, after the proposed restrictions in 2020^(27)^ but before the UK government’s consultation response^(28)^ (and thus prior to the Act’s passing in 2022 and before decisions made by UK Governments to delay the legislation coming into force until 2025). They ranged from 30 to 75 min and were conducted online (on camera where bandwidth permitted) and audio recorded.

MTG and EB, in consultation with ZH and VT (who provided feedback on the draft questions), developed a semi-structured interview guide covering seven key issues regarding policies to regulate digital marketing of food and non-alcoholic beverages, including policy scope, awareness of other existing policies, interactions with existing policies and regulations, roles/responsibilities of key actors, unintended consequences, monitoring and enforcement and future-proofing (see online Supplemental Material). All experts were introduced to this full scope of the study, but given experts’ limited time availability, the interviews focused primarily on each individual’s area(s) of expertise (interviewees were invited to identify any questions which went beyond this) and on the issues that they, as experts, considered of greatest relevance. All interviewees were asked about the scope of the online restrictions proposed in 2020^(27)^ in a consultation for the upcoming Health and Care Bill; aspects of monitoring relevant to their expertise; the impact of marketing and the proposed policy on health inequities and future-proofing the regulation. When time permitted, additional questions for marketers and marketing specialists focused on marketing design and strategy; technical experts on digital marketing delivery and monitoring and obesity and regulatory experts on national and international regulation and policy. At the end of the interview, experts were invited to provide further commentary on additional issues they considered to be pressing.

### Data analysis

Audio recordings were transcribed verbatim. Researchers conducted thematic content analysis^(38)^ of the interview data to identify meaningful and relevant expert views of the UK proposed legislation, including its potential impact, and to construct a coherent narrative account. Deductive analysis was based on predefined high-level categories drawn from the interview topics; through inductive analysis, led by the fourth analyst (JH) who was external to the data collection process, the research team developed themes responding to further content in the dataset^(38)^. This iterative, consensual approach followed principles of Consensual Qualitative Research (CQR)^(39)^; CQR’s main features are collecting data via semi-structured interviews with open-ended questions; taking multiple perspectives by having multiple researchers carry out analysis; arriving at consensus about interpretation; using a further researcher who was not involved in data collection to review the analysis and reduce ‘groupthink’ and identifying core ideas and domains in the analysis. Deductive themes were first identified by MM (using NVivo version 12 as a tool) and reviewed by MTG, cross-checking against the full transcripts to ensure they reflected the range of perspectives covered in the interviews. Inductive themes were then developed led by JH. Finally, EB and MTG reviewed the themes for overall coherence. At each stage, differences were resolved by discussion to achieve consensus. The content analysis themes developed reflect the range of views expressed across the expert group and are not necessarily reflective of the view of any one participating expert.

## Results

In the thematic analysis, researchers identified four overarching themes with twelve deductive and inductive categories (including seven sub-themes) regarding overall assessment of the originally proposed UK legislation, and key issues related to policy design and implementation, monitoring and enforcement. The four major themes spanned, first, an overall assessment of the UK online restrictions that would later be adapted to become the Health and Care Act (2022). Under discussion was the online advertising ban as originally proposed in 2020^(27)^ but prior to the 2021 government response^(28)^: first, its novelty and limitations; second, the design of the policy, in terms of its scope at the time of interview, the ages it protects and alternative policy options; third, policy implementation, with a particular focus on tracking and monitoring; and finally, the wider picture, considering the overall advocacy agenda, coherence with other policies and social norms regarding unhealthy food (see Table 2).

**Table 2.** Themes and sub-themes identified from experts’ views of the UK online digital restriction policy

I. Overall assessment of the ‘total’ online restrictions as proposed in November 2020^(27)^ for the subsequent UK 2022 Health and Care Act
Ia. Novelty: ‘A potential major breakthrough’^*^
Ib. Inequalities and child rights: ‘This is a profound matter’
Ic. UK policy limitations: Being ‘brutally honest’^*^
II. Designing the policy
IIa. Scope of the policy:
1. ‘Why are we excluding brands?’
2. Additional current digital marketing ‘the immersion of content and advertising’
3. ‘The problem is going to get bigger rather than smaller’
IIb. Ages to be protected: ‘The total ban [is] much, much better’^*^
IIc. Alternative policy designs: ‘Needs to be ‘no’, by whatever format’^*^
III. Policy implementation, monitoring, and enforcement
IIIa. Digital advertising ecosystem: ‘No transparency’^*^
IIIb. Tracking and monitoring:
1. ‘They should be proactive’
2. Complex systems and technical challenges need ‘high-tech solutions’
3. Feasibility: ‘You go and figure it out. You have got billions’
4. Other types of monitoring: ‘Link an action to an indicator’^*^
IIIc. Regulatory structure
1. ‘How are these going to be enforced, who is going to be responsible?’
2. ‘It has to have some bite’
IV. The big picture
IVa. Advocacy agenda: ‘Coherence of rules is important’^*^
IVb. Interaction with other policies: ‘An opportunity on the European level’
IVc. Social norms: ‘We are slowly moving there’^*^

* Inductive themes.

Experts applauded the UK restriction as originally proposed in November 2020^(27)^ (including online paid and owned HFSS product advertising; see the online Supplemental Materials for further information) as a major step forward in advancing public health and addressing socio-economic inequalities. Yet they also identified numerous factors that would likely limit its potential impact. Their comments focused on the design of the UK law and other potential policies, as well as wider issues of policy implementation and processes needed for effective monitoring and enforcement.

Note that ‘owned’ marketing (i.e. marketing on brand and product-‘owned’ social media accounts) was in scope in the original 2020 proposal^(27)^ and when these interviews were conducted, but the government’s 2021 response to the consultation concluded that ‘the new restrictions will not apply to an advertisers’ owned media (website, apps, social media page, video sharing platform page and so on)’^(28)^. Therefore, owned media were not included in the final UK Health and Care Act (2022), along with some further adjustments^(28)^. Thus, compared with the original 2020 proposed version interviewees discussed, the UK government’s final 2022 ‘total’ ban is weaker.

### I. Overall assessment of the online restrictions as originally proposed for the UK 2022 Health and Care Act

#### Ia. Novelty: ‘A potential major breakthrough’

Interviewees considered the restrictions to be novel. They knew of no other existing policy with a similar aim or reach and described it as ‘exciting’ and a ‘great achievement’. Most viewed it as a major step forward for the health of the UK as a whole, with the potential, as a statement of intent, to lead globally by providing a positive example:

*What you have proposed in the UK is a potential major breakthrough in terms of public health in the 21st century [6]*

*There is a huge great gulf there, in [regulating] what is unquestionably the biggest, but arguably the most powerful and most effective form of advertising. [1]*

#### Ib. Inequalities and child rights: ‘This is a profound matter’

Experts also agreed that any regulation addressing HFSS food marketing (including the online restrictions as proposed in 2020^(27)^) would certainly address socio-economic inequalities, which are fundamental to this public health issue. In doing so, they focused on the underlying concern that highly processed foods are marketed to, targeted at and eaten more by less advantaged social groups across Europe and the USA. Therefore, any regulation of unhealthy food marketing would in their view benefit these groups the most. However, experts did not refer to any specific features of the digital restriction itself in their responses on this issue.

*A lot of this advertising of unhealthy food and drinks is not for the wealthiest in society necessarily, right? A lot of it, it’s because the food is cheaper than healthy food so you’re targeting it at lower-income people [3]*

*In the United States the reason to regulate is precisely because of the now commitment to address inequities and inequalities that communities of colour in the United States, and … the statistics there in the UK, have higher rates, children of colour and youth, of these health-related issues and also are heavy users of digital media. So the need to regulate is really being pushed by the need to ensure there aren’t inequities in the public health system. [6]*

*The socio-economic division of the distribution of obesity in this country [UK], there is clearly a factor that the more disadvantaged you are, the more likely you are to be carrying a dangerous level of weight. … So, from literally medical outcomes to a sense of identity, being, and confidence in yourself, this is a profound matter. [8]*

Some experts also placed the issue in a child rights context, noting that under the United Nations Convention on the Rights of the Child (UNCRC)^(40)^, which the UK and all other countries except the USA have ratified, children have rights not to be economically exploited. Therefore, children’s best interests are ‘of a higher order than commercial considerations’ [Interviewee 8]. These experts argued that children have the right to be removed from online advertising’s privacy-breaching ‘extractive business model that is commercially exploiting them’ [Interviewee 8].

#### Ic. UK policy limitations: being ‘brutally honest’

Having identified the novelty and significance of the policy in establishing the principle of restricting HFSS digital marketing, at the same time, experts also predicted that the proposed UK policy would be largely ineffective – and indeed, in the case of one interviewee, ‘entirely’ so. Marketing experts predicted that marketing activity would shift from techniques that are banned by the policy towards other, non-regulated forms of marketing. In particular, they noted that the policy by definition would only restrict marketing with depictions of actual HFSS products (i.e. with products that could be assessed and identified as not meeting nutrient profiling criteria in the current proposal). This would lead companies to focus even more on brand marketing instead: promoting a brand, but not featuring a specific product offered by that brand:

*If I am brutally honest with you, the regulations that are going through at this moment in time will for the most part, be entirely ineffective. They are great from the point of view of principle, but what you are going to see is a significant growth in brand advertising rather than product advertising… I am terribly sorry to say that about these regulations because they are exciting, and it is a great achievement. [1]*

*I am very happy that UK is taking this step [but] … if we just take the burger from the picture, I am not sure what are we really banning. … Although it’s very good, to not allow showing unhealthy food products, … brands continue generating those close relationships with children and adolescents, [so] probably… we are not getting the effect we are seeking. [5]*

Experts also provided recommendations to address additional limitations in the scope of the UK policy and to establish greater monitoring and enforcement mechanisms, which are described in the next sections.

### II. Designing the policy

When discussing optimal digital marketing policy design and how to future-proof policies, experts argued that effective regulations must cover the full range of powerful, widespread digital marketing techniques that promote HFSS products and/or their brands.

*For very little money, these [digital marketing] techniques work. … There is a list of techniques and practices that need to be regulated along with the problematic product categories and their use in brands. If you are using artificial intelligence and machine learning and data-based profiles, influencers, virtual reality, augmented reality, geolocation, tracking and targeting… The range of techniques. These techniques should not be used to facilitate the brand marketing of products [6]*

Experts highlighted the need to cover brand marketing and other marketing techniques in the regulation, as well as other important design considerations.

#### IIa1. Scope of the policy: ‘Why are we excluding brands?’

Interviewees all agreed that brand marketing must be covered by the regulations and that restricting only marketing of HFSS products was a major loophole in the UK regulation. Experts commented extensively about the power of the brand and the expectation, as noted above, that HFSS product advertising would simply switch to brand advertising. Interviewees explained that embedded throughout all marketing strategies is the brand and that brands are always associated with products.

*There are obviously question marks around brand advertising. [1]*

*I think a very important thing is to start to discuss why we are excluding brands [from the regulation]? … one of the important things that we did in Chile was stop talking about trademarks. We are talking about advertising. Whatever that is. We want to restrict advertising of unhealthy food. … The brand has an advertising function so if it is targeting children, it is under the scope of the regulation. …why are we giving to intellectual property a level that is higher than a public health regulation? it is not clear. [5]*

*There is no empty brand. A brand always has products behind… I mean the brand will not exist if there is not a product related to it and if I don’t talk about which product I am promoting, so: you are promoting all of them. [5]*

Moreover, experts stated that brands are designed to evoke emotions and values. Companies’ ability to use marketing to associate brands in young people’s minds with crucial developmental needs would continue. In particular, common digital marketing techniques take advantage of adolescents’ developmental needs to establish their own identity and to foster strong peer relationships.

*You’re talking about these deeply embedded, cultural, psychosocial values around the brands and product categories that are far-reaching. [6]*

*Brands are used to develop the self-concept, are used in the process where we are developing our identity, so this symbolic meaning, and the social meaning of a brand, it is crucial for adolescence. And in the digital world, food marketing brand marketing is used to develop and is used to communicate with their peers in the social space … that is very important. [5]*

#### IIa2. Scope of the policy: additional current digital marketing ‘the immersion of content and advertising’

The UK policy as originally proposed in 2020^(27)^ and discussed by interviewees in 2021 primarily addressed marketing in paid media; advertising that companies purchase in digital media (e.g. online display ads, paid searches, email/text messages); marketing for which the company has created or contributed to the content (e.g. brand websites, social media pages) including owned media (which was excluded from the subsequent 2022 Act) and paid influencer marketing (see online Supplemental Materials for full list). Interviewees however identified many additional digital marketing techniques that do not qualify under the UK 2022 online restriction but currently form a key part of the marketing mix, including advertainment (e.g. gaming, influencers, product placement), sponsorships, corporate social responsibility, online retail and data usage techniques (see Table 3 for experts’ views on these out-of-scope marketing techniques).

**Table 3.** Expert views of the power of digital marketing techniques that *do not qualify* as in-scope (i.e. purchased advertising or food company-initiated marketing for HFSS *products)

**‘Advertainment’** Advertainment often includes brand marketing embedded within entertainment content (e.g. games, movies, TV shows, music) and/or viral marketing that is created by third parties and shared virally (e.g. YouTube influencers, user-generated social media posts). Brand marketing in games – as the experts discussed elsewhere – allows marketers to bypass a product-focused ban **Gaming** *The food and beverage companies, alcohol companies, talk about an area of how you stay ahead and where we missed… …gaming is bringing in more revenue than motion pictures. Gaming is increasingly ad-supported or ad-facilitated. [6]* **Influencer marketing** Experts also identified blurred lines around influencer marketing – where the brand–promoter relationship may be unclear *I think it’s so important to focus more on the content marketing which I realise is far more difficult … because the lines are so blurry between normal content and promoted content, but …from what I can tell, if you have an influencer, that is identified as a big brother or a big sister, and they talk about all these great products, I think that is a lot more effective than … targeting based on millions of data points to try to get you buy Coca-Cola, for example. So I really think that they have to spend more time looking at content marketing, influencer marketing. [3]* **Digital product placement** They also highlighted the complexities raised by new digital advertising options within programmes and even within content itself *You can put an ad into a Friends episode and put a new product in there for sale… Companies … are doing in-game banner programmatic advertising. They can sell the advertising in a FIFA game, to build a real-time programmatic ecosystem around that. Obviously, audio comes into it a little bit. That is today, that is not tomorrow, right? [2]* ***Other types of marketing*** Experts specifically mentioned a few other types of marketing that would not be covered by the UK policy. Although sponsorships are included in the proposed regulation, most sponsorships promote a brand and not a specific HFSS product, according to experts **Sponsorship as brand advertising** *Sponsorship, which seems to be growing in popularity, particularly in sponsoring and creating content around that sponsorship, and distributing that content through social media, is a big, big growth area in brand advertising now. … I think that is pretty much totally absent from the regulations …, because it would tend to be very brand led, and it is sporting events and things of that nature, which do have a very wide appeal, including children. [1]* **Corporate social responsibility** *The net impact of most CSR is absolutely minimal, and almost all CSR is being done because it has a direct return on investment for the company there and then [7]* **Online retailers** *Grocery tech, the role grocery stores play. You [UK] have been in the lead … your big chains. … Tesco and all of that, are also data and advertising companies and work with the brands to target families through digital coupons and other incentives [6]* *I think we [Chile] need to take care of that and now, because we have a monitoring on TV ads, we have seen also that the ads have - for instance of course we have the delivery apps here - have also increased a lot, …- or this delivery app a shows a pizza, it’s not really a pizza ad but it makes you feel like having a pizza, so again… [4]* **Data techniques** Online advertising utilises a wide array of user data, including individual characteristics and behaviours and geolocation, to target advertising messages *The techniques used in the campaigns need to be incorporated in the scope, because the techniques in the campaign are designed to further sales of the key product lines of the brands but also to collect more data for targeted marketing subsequently. [6]* *The delivery companies are partnering with the junk food companies, sharing data, and engaging in joint targeting. They would have to be included in the regulatory scheme. I don’t know about the delivery companies in the UK, but certainly in the United States… Uber Eats and all that. [6]* *If in fact you are using artificial intelligence and machine learning and data-based profiles, influencers, virtual reality, augmented reality, geolocation, tracking and targeting… The range of techniques. These techniques should not be used to facilitate the brand marketing of products that are known to be of interest to use [6]* **Owned media** Note that experts did not comment on the absence of ‘owned’ media from the list of restrictions as the original proposed policy they were addressing encompassed this mode of advertising, whereas the final 2022 Act did not.

*HFSS, high in saturated fat, salt and sugar.

Many of these additional digital marketing techniques consist of brand messages embedded within entertainment content, also referred to as ‘advertainment’. These less-traditional forms of marketing, such as brands or products featured in influencer videos (whether explicitly paid for or not), product placements, and in-game brand marketing, blur the line between advertising and entertainment, enabling advertisers to grab users’ attention in the crowded online advertising space and connect with them emotionally.

*The blurring of lines between advertising and content, it is not a futurecast. It is present. That is the story. That is what is happening. I think we need to be terribly conscious of that. … I suspect the high street will become increasingly more about content and experience, rather than product. That is the way it is all going, the immersion of content and advertising. Creating great experiences is how you sell people your product, ultimately. Creating affiliations to their passion points is how people are going to sell products. [1]*

Experts frequently mentioned *gaming* as an important current and future venue for digital marketing. They pointed to in-game marketing encouraging consumers to actively engage with brands rather than passively viewing the ads delivered to them.

*Gamify the marketing to an extent where you can’t really tell the difference between a game and an advert … moving into things where kids are actively seeking out advertising because it’s a game… ads are going from being this necessary evil that you need in order to get something for free, to become something that you’re actively seeking out, and I think that’s a bit of a change [3]*

*The brands are holding these virtual concerts, these huge, public promotional events in these gaming platforms that reach tens of millions of young people, many, many more young people… this is once again very important in a post-pandemic world, these virtual, collective, national, regional, global events like concerts and e-games, sporting events, supported by the food and beverage companies that reach tens of millions of young people. [6]*

*It’s this kind of blurring the lines even more, between content entertainment and advertising, … we’re seeing perhaps the first seeds of this in a fully-fledged way for the video game Fortnite, for example, where they’re trying to brand themselves not as a video game but as kind of an experience generator; … you can show movies, you can have concerts and they’re basically combining it with the social network and you have brand takeovers and so on which is of course present in other platforms as well, but they’re trying to make it a more immersive experience. [3]*

Some experts also mentioned types of marketing (e.g. sponsorship) that were technically included in the scope of the UK regulation, but in most cases feature a brand rather than a specific HFSS product that could be subject to nutrient profiling – and therefore the advertising would be permitted. They also mentioned CSR marketing, as well as marketing that occurs during online food purchases through grocery stores, other e-commerce and delivery services as an often-overlooked venue for digital marketing. Finally, experts discussed data-driven approaches that are used to amplify marketing effectiveness, such as through analysis of consumer characteristics including location, and campaign impact, as a form of marketing that must be included in regulations.

Interviewees also argued that more traditional types of online advertising (e.g. banner ads, company websites) covered by the UK policy would become increasingly less effective as advertisers compete for attention in the increasingly crowded digital advertising space.

*The digital sphere is so commercialised that you have advertising everywhere constantly and … advertising banners and so on, I think that entire industry has eaten itself because you’re being bombarded with ads so the attention… Nobody I think really looks at those ads and I think our brains are filtering it out because there’s too much noise. [3]*

#### IIa3. Scope of the policy: ‘The problem is going to get bigger rather than smaller’

When discussing how to future-proof regulations, experts disagreed on virtual reality (VR)’s potential to amplify marketing impact. Two considered it to be a well-developed present-day technology that will become increasingly meaningful:

*They were like, ‘That’s in the future.’ It’s really not. … Just going on a holiday by having an experience, rather than actually physically going there, that is closer to reality now than it has been before, both in terms of consumer demand and the technology to do it. [2]*

Another expert expressed doubt that VR would gain extensive traction, given that it required expensive technology such as headsets, which was not within the means of less-advantaged groups that HFSS advertisers particularly aim to reach:

*I’m personally skeptical about these claims that you’re going to see virtual reality being the next big thing… they’ve been saying that for decades and it’s not something that everyone has access to … trying to put too much effort into making advertising campaigns on VR glasses for example, that’s not going to happen because the people you want to target aren’t using that technology, they don’t have access to it. [3]*

One expert predicted that the growth of in-game marketing would extend into virtual and augmented realities in the coming ‘metaverse.’

*You hear of the metaverse, which is literally, we are all living in the computer game, in Ready Player One. It is like the next generation of what Augmented and Virtual Reality will be… The metaverse will become this never ending self-generating extendable universe of content. A lot of work is being done on the computer processing side that is required to have ultimately billions of people online at the same time having digital interactions rather than physical interactions. Then the currency and the experiences around what happens in the metaverse around commerce is also super interesting and key. [2]*

*This side of the economy is going to grow quite quickly … the sad thing is that our grandchildren are probably going to be plugged into the wall 50 % of their lives. It is already happening with education, so why isn’t it going to happen everywhere else?… You can have someone who just comes and talks to you in the metaverse, and sells you a product, a purely digital influencer. It doesn’t have to be a banner ad or something. So, I think there will be a lot of innovation in terms of, how do brands participate in the metaverse. [2]*

As a result, this expert anticipated that marketing will become even less trackable.

*Well, if you think about traceability… I have no way of knowing now, when [my son] is playing FIFA or whatever he plays, Call of Duty, to know what brands are advertising to him, no idea. The numbers of platforms and the number of experiences, and the different programmes that you are going to be interacting with, the problem is going to get much bigger rather than smaller. [2]*

#### IIb. Ages to be protected: ‘The total ban [is] much, much better’

Experts agreed that the UK complete (all ages) online ban on HFSS marketing would be much more effective than existing HFSS food marketing regulations that focus on children’s programming and/or child-oriented content.

*The total ban serves [the purpose] much, much better. [7]*

This belief was based on both technical and ethical reasons. Technically, it is challenging for digital providers to identify the age of any given user, even when platforms have different versions intended for children or older users.

*You get into these age breaks, and that becomes tricky, because without having that digital age verification at scale embedded in the system, we are down to separation from publishers’ perspective. So, you can have a certain set of rules for YouTube, but a different set of rules for YouTube Kids, but you will never know if a 13-year-old is watching YouTube, which of course they are, instead of YouTube Kids. It is that separation which is quite challenging the way that it is working today. [2]*

*If targeting, other technology, or other limitations mean you can’t differentiate between a child and an adult, then default positions should be that the marketing does not go ahead. If not otherwise known, you should assume the person is a child. [1]*

The only other solution would be complete age verification of all internet users, and experts were uneasy about this as it could negatively affect children’s *privacy* in order to protect their *health*. Although one expert predicted that the overall drive to protect children from harmful internet content meant that in the end, complete and mandatory UK age verification was inevitable, others argued that maintaining privacy protections remains critical. Overall, experts believed that the all-ages approach applied in the UK regulation is preferable to collecting more data from children.

*I would say the endgame on this is that in order to use the internet in the UK, the government requires you to have a verified ID and you have to log onto all services. At that point, the content and the advertising that is served to an individual can be restricted based on age. But as long as you don’t need to have that verified ID to get into the services, then you can just set up an account and tell anyone that you are 21. [2]*

*I don’t want to be in a place where every single website is trying to age-gate people …. That’s way too much data-gathering in order to protect people from the dangers of data-gathering, it doesn’t make any sense. [7]*

*I know that this is something that’s been talked a lot about in particularly the UK about this kind of real identity policy online…, which I think is a horrible idea which will only harm the people who need anonymity and which are the most vulnerable populations normally [3]*

*That is something we need to learn. How we can increase protection of privacy at the same time that we have the tools to restrict targeting content? [5]*

Another benefit of the UK all-ages approach was that it eliminates a loophole identified in other regulations of marketing to children: family-based marketing. In Chile, brands moved to targeting parents and families with marketing messages for children’s foods.

*And we have also seen a shift of course … maybe also mostly like for mothers, like a ‘love of mum’, or ‘what is better for your child’, so it’s not a child target - but of course finally it is! [5]*

*This is where it gets tricky – how there are so many ways to market, reach the child without directly targeting. Family-based marketing is incredibly effective, let’s call it community-based marketing, so they can easily market these foods to the mums and dads when they’re in the local store, giving them discounts right at the moment, selling the products they want, the use of influencers allows them to not directly advertise. So in order to think about stopping the proliferation of these potentially harmful products, reaching the UK’s youth, one has to think beyond the age-based guidelines and safeguards I think. [6]*

Furthermore, experts highlighted that the powerful persuasive tools available to digital marketers today also affect adults, again concluding that implementing a ban for all ages would be more meaningful. Moreover, adult-directed content also affects children.

*The tools that we have today to communicate persuasive content are very complex, are very strong… this is not a problem of children, and it’s not a problem of rational thinking, it’s not a problem of intelligence, it’s not a problem of development. Of course, there are some development issues that make some communities or groups vulnerable … impulse control and identity processes may make adolescents very vulnerable - but probably an adult that is under stress with a lot of cognitive load is in a similar position of vulnerability. [5]*

*My study … was actually [looking for] a differential effect between child-directed and not child-directed ads promoting exactly the same product – and there is no difference. So we are so happy banning child directed content, but the same emotion, the same appeal with adults, has exactly same impact on children. [5]*

#### IIc. Alternative policy designs: ‘Needs to be ‘no, by whatever format’

During these interviews, some experts also proposed alternative approaches to regulating digital HFSS marketing that could help to close loopholes and future-proof regulations. For example, one expert proposed a reversal of the typical food marketing regulatory process: instead of using nutrient profiling to identify food items *not* permitted for advertising, a system should be developed that only *permits* food marketing if the product and brand demonstrate their nutritional benefits.

*The absolute core substance needs to be ‘no,’ by whatever format; whether it is paid, whether it is earned, whether it is influencer. Sitting behind that, every single one of those, there is a brand who has some control over their product. [1]*

Adult opt-in to receive HFSS marketing content, such as emails, grocery offers and similar forms of marketing, was also suggested as a feasible option. This would take the onus off children to report their age and to opt out of harmful marketing.

*So, if we are doing an opt-in, we are placing the responsibility on adults to opt into things that are potentially harmful…. But it’s really important to think through what we’re doing with opt-out systems. With opt-out systems, we have basically created this piece of dangerous infrastructure and then we are requiring children to extricate themselves from it by proving who they are, providing some information, so we are putting the responsibility on children … placing it on the shoulders of children to protect themselves from this dangerous infrastructure that we have built [7]*

However, opt-in approaches would require greater honesty and transparency in advertising and labelling to ensure that brands desist from making claims that are, in effect, misleading.

*I think there are interesting question marks about where people have opted in, to receive information, particularly for adults, if they sign up to email lists, for example. [1]*

*I think there needs to be a little bit more honesty and transparency in advertising. There is too much advertising that effectively misleads. For example, you might see cereal advertised, and they will talk about something being very high in fibre, but there is actually no great transparency in the fact that it is also very high in sugar. I think if we make sure that there is very strong front-of-pack labelling, possibly in advertising labelling for products that are high fat sugar salt, then I think businesses should be free to advertise in a way that is honest and transparent to adults. Adults should be free to choose. But I think it is good practice for society to make sure that choice is an informed choice, and not an uninformed choice [1]*

One expert took the view that, logically, if products’ availability and distribution was not restricted, advertising of such products should be permitted. This expert also raised concerns about a potential economic impact for advertisers of the regulation post-pandemic.

*In a post-pandemic hospitality sector, you are cutting off a lot of economic activity …I think from a policy and a regulatory perspective, you can’t just look at media as the problem and online exposure as being the problem, if distribution is as much of a problem.…My only caveat there would be, there is no point restricting the marketing if you are not restricting the distribution. [2]*

Overall, experts were skeptical that digital marketing regulations could fully eliminate HFSS food marketing online. Given the slow pace of governmental regulation, interviewees agreed that industry and marketing will already have outstripped the regulation when it comes into force, by developing new methods and marketing techniques. They argued that a high-level, principle–based approach to future-proof regulation was necessary due to the complexity of the digital advertising ecosystem and its constant cycle of rapid, adaptable invention.

*Advertising money is like water. It will find a way to get to consumers, no matter what you try to do to block it. [2]*

*Trying to get it up to the right level so you are actually describing what it is you want to stop, versus going down into the more detailed kind of technical solutions … that’s really important that the legislation is framed at the right level. [7]*

### III. Policy implementation, monitoring and enforcement

In addition to challenges in designing and future-proofing digital HFSS marketing regulations, the experts also identified numerous issues related to implementing, monitoring and enforcing such policies. The global nature of the advertising system presents an important challenge.

*Where the problem is going to be with that is the international nature of the beast, that most of the kids, the influencers they are watching are based in the US, and the technology that hosts them are based in the US, and they sit outside the jurisdiction of the UK. That is going to be quite a challenge, and that will just become more so. Ultimately, at the end of the day, if I wanted to, I could put all my servers in Sierra Leone and do anything I like, right? [1]*

#### IIIa. Digital advertising ecosystem: ‘No transparency’

Unique aspects of the digital advertising ecosystem also present implementation challenges, including the dominance of major platforms globally, such as Google (includes YouTube), Meta (includes Facebook, Instagram) and Amazon. Experts described these platforms as ‘walled gardens’ in terms of oversight.

*The other impact… is the growing share or participation of Google, Facebook and, to some extent, Amazon, in the overall digital media ecosystem. The fact that you are effectively talking about controlling or guiding the management of the Facebook and the YouTube platforms, which probably is where 85 % of the issue is [2]*

*The big thing about Google now is that, with this CMA [Competition and Markets Authority] report on understanding the ad technology stack penetration of the Google ad stack, in addition to YouTube, in addition to Gmail and all the other Google services, they have got a very, very dominant position in ad-serving and in demand-side platform buying and on publisher platforms. … If there is ever to be any regulation of Google, it will be the separation of that ad technology business from the publisher business. [2]*

Experts also were of the view that the current digital advertising ecosystem allows a diffusion of responsibility, with brands, advertisers and platforms all claiming not to have any oversight over ad delivery.

*A lot of this accountability and responsibility is completely pulverized. [3]*

*Digital is bigger than everything else put together, but there is effectively no transparency at all. Nobody has any idea about who has seen what, and what is out there. And there is no form of, not even the lightest touch level of approval, certification, regulation or anything that might protect it, other than where something is reported to the ASA [Advertising Standards Authority], and the ASA can amend their rules. [1]*

#### IIIb1. Tracking and monitoring: ‘They should be proactive’

All experts agreed that outside monitoring of digital HFSS food marketing is required and must be ‘proactive’ rather than reactive or complaints-based.

*I am always going to say they should be proactive. [1]*

*I would definitely say they should be proactive. I think what we use so far and what we see in our work, it’s just this kind of reactive enforcement; it’s really time-consuming. [3]*

#### IIIb2. Tracking and monitoring: complex systems and technical challenges need ‘high-tech solutions’

Many interviewees observed that the automated programmatic nature of online ad buying systems makes it particularly difficult for outsiders to monitor what ads are being seen and by whom. Some pointed out that automated monitoring is useful, such as regular automated websites sweeps carried out by the UK’s Advertising Standards Authority to identify child-directed advertising, but it has limitations. In particular, this type of monitoring cannot enter websites that require sign-in nor assess ad exposure^(22)^.

*Yes, I think there is a way to monitor it, but it is a high-tech solution. I think it sits very much outside of the skills and culture of the Advertising Standards Authority, which was built for a different era and doesn’t really think in the right way or have the skills within its organisation to respond to this. There will need to be a technical solution that is using some kind of monitoring bot. Again, I go to think that there are certain environments, and unfortunately, they are the biggest environment that prohibit monitoring bots from coming inside them, such as Facebook being a good example. But I think the impetus that comes to them, that says, ‘If you want to be free to advertise these kinds of products, then you need to be open to some kind of moderation.’ [1]*

In addition, some experts believed that even the platforms and the advertisers may not know precisely where their ads are placed and who is viewing them.

*If I go to the trade desk and book my campaign through my advertising agency on the DSP [Demand Side Platform], and I am Coca-Cola, I can say, ‘I want to target 18–35-year-old females, but I only want each individual to see the ad 5 times or 10 times in the campaign.’ You can set that up. What actually happens will be, on average, you will hit that, but the measurement is not perfect. There will be ads that are shown on publisher sites where that woman is not logged in, so she will have more impressions than she is actually being measured for… all within the inner workings of the ad tech ecosystem and how that is all wired up. It is possible to control, but not to 100 % determine exactly what happens… [2]*

*Facebook knows exactly the ads that every account sees within their publisher universe, but that is within their walled garden. It is across the whole internet that is quite difficult… We were just running a campaign last week. The technology platform thought it had delivered three times as many ads as the publisher said they had delivered. That sort of automatic ad format by ad format measurement of delivery is still something which has got loads of leaks in those pipes. You would never be able to, on an individual level, I think it would still be hard to work out. [2]*

*Despite spending all of this money, [global confectionery brand] didn’t really actually know who was seeing their ads, and so actually they had very little idea, or they didn’t have actually the really granular picture that people think you get from digital marketing campaigns, where you can know exactly who has seen your ad. [7]*

#### IIIb3. Tracking and monitoring feasibility: ‘You go and figure it out. You have got billions’

Experts expressed differing opinions about whether monitoring compliance was needed. Some noted that the major platforms have a reasonably accurate understanding of who is exposed to the advertising placed on their sites and believed that monitoring digital marketing was technically feasible.

*There’s no doubt in my mind that …the technologies and methodologies are out there increasingly, so you can make sure [specified foods] aren’t targeted certainly to youth. [6]*

*I am not going to get into technical conversations about this, this and the other. You just don’t do it. We need to say, ‘You don’t do it.’ You don’t advertise unhealthy food to children. You go and figure it out. You have got billions. You have got the cleverest technical people in the world. You have got amazing infrastructure. Go figure it out. If you can’t figure it out, don’t do it. The basic principle is that it should not be okay to promote the consumption of high fat sugar salt products to children… [1]*

Some discussed examples show it is possible to build systems to control content placement on the internet when a commercial imperative exists, such as copyright law, or brand safety pressures that prevent ads being positioned next to unpleasant, socially unacceptable or dangerous content.

*Google and Facebook can tell us pretty much, of the 60 % of authenticated users that they have at any point in time, with high degree of accuracy, these are the ads that have been received by them, and these are the ads that have been responded to by them. But that is part of the beauty of those platforms, that they have that level of accurate information at scale, that they can then sell to advertisers and say, ‘Here is the proof that your advertising is effective.’ [2]*

*So, when it comes to copyright … Everything is technically possible and regulation exists to make it so… There is copyright law. And so, I think, again, if you say is it technically possible, is it regulatorily possible, yes, yes. Is it happening? No… Not on a fair terms basis … this is the same community that are thinking they can take us to Mars, can cure all ills through data processing and so on and so on. [8]*

At the same time, others argued that existing content monitoring systems are flawed, and such regulations could have unintended consequences.

*Where you want to hold platforms responsible for copyrighted content and want to have them use these automated technologies – what we call filters – to take down things that might breach copyright, and then it turns out that maybe these technologies aren’t able to tell parody or satire, for example. And we really wouldn’t want that in the name of preventing content advertising; we don’t want an internet that is pre-filtered. So, I think you have to be cautious about how you use these technologies and never over-rely on them. [3]*

However, most agreed that effective implementation and monitoring would require the cooperation of the large platforms, even while questioning their incentive to build and maintain transparent systems that could allow outside monitoring of the advertising on their platforms.

*I think you come to responsible practices on behalf of the majority of the platforms. If, with a high probability, things are definitely not passing the nutrient profile test, then they shouldn’t be advertised, and there is a high probability that someone is actually under 16, they shouldn’t be advertised to with a longer list of products. But it is requiring some good actor behaviour on behalf of Google, Facebook, Snapchat, Twitter. [2]*

*Not without a very strong top-down commitment to clean up Facebook. There are activities, forums and policy units and everything that can be set up to pay lip service to a problem. But if the economic incentives aren’t aligned with clearing up the problem, then I would put my bet on the fact that the problem won’t get cleared up very quickly. [2]*

As evidence, some experts pointed to platforms’ weak performance regarding self-regulation. For example, Google has implemented an internal ‘no advertising to under-18s’ HFSS policy for the UK and the EU. However, it relies on users to accurately self-declare their actual age to restrict delivery of ads and can only be applied when an individual has logged into a Google platform (e.g. YouTube).

*Transparency measures from the big platforms where you can go and click ‘Why am I seeing this ad?’ and so forth which has, at least to me, been completely useless because they don’t give you any information that you can actually use. [3]*

*Google is another really interesting one. So, again, that’s another ‘voluntary’ stance by the platform, but as soon as you look into the implementation of it, its effectiveness falls away so quickly… they would no longer show high fat, sugar, salt adverts to those people who are identified by the platform as being under-18, and this was identified by the platform by their self-declared birth date, and we know that there’s a huge amount of age lying that goes on when people sign up to YouTube. [7]*

*Technically, if you are a logged-in user onto YouTube, or onto the Google universe, and you have said that you are under 18, they have the technology to negatively target any ad campaign for any restricted food category so that you never receive that ad. That is 100 % deterministically possible. It is just that … A lot of the usage is not when you are logged in, number one, and secondly, that most people who have told you their age, tell you the age that they need to be to get into a restricted category content. [7]*

#### IIIb4. Tracking and monitoring: ‘Link an action to an indicator’

Finally, some experts argued for additional monitoring to assess indicators of impact, not just exposure to digital HFSS food marketing. These indicators should be tied to the actions taken. One US-based interviewee recommended annual or bi-annual public health audits performing ‘digital health surveillance’, with an obligation on governments to act to address inequalities, realise rights and facilitate implementation and monitoring.

*It’s very hard to see an effect … of a marketing regulation on BMI [body mass index] or obesity reductions …maybe that has been also [our] mistake that we didn’t link clearly an action to an indicator. So, for instance, if the action of prohibiting or regulating marketing Internet would be decreasing by 10 % the obesity rate in children under 5 year old in five years -- to succeed is impossible. So which are the indicators? Maybe the indicator is that the ads are no longer there. Or the indicator is that, if you ask a child, they don’t remember the X brand, or whatever or they don’t have a linkage, an emotional link, or fidelity, loyalty to the brand or whatever. [4]*

*Something specific, outcome, must be prevented or must be enabled or so on and so on – it has to be precise. It can’t be ‘something must be done’. [8]*

*That’s where public health audits come in. … the companies regularly report the data, the data systems are open to review in part, the public health experts review the data on an annual basis and determine the effectiveness of the regulation, what else is emerging. … It seems to me that this is a place where the public health infrastructure, Public Health England, Public Health Canada… It would be great if our FDA [Food and Drug Administration] did this here [in the US]. It needs to create a system on digital health surveillance to understand the impact of these technologies and applications… In the United States it’s not been as effective as they would like, but the civil rights groups for Facebook and a few other platforms, Airbnb, to do an annual civil rights audit… companies already have to do these audits for the GDPR and data protection compliance, so there’s a methodology and tradition out there that’s growing. More and more people are calling for these audits to be made, to be accessible to regulators, and then to be acted upon. [6]*

#### IIIc1. Regulatory structure: ‘How are these going to be enforced, who is going to be responsible?’

Experts agreed that the regulatory structure is critical. It must be well-defined with strong mechanisms to enforce compliance. Co-ordinated activity across regulators, including a clear lead, is required to achieve regulatory oversight of this extremely complex advertising ecosystem.

*You do need that regulator, just to focus the attention, and to have an interface to collect the issues. [7]*

*How these are going to be enforced, who is going to be responsible, are … almost as important questions to think through at this stage rather than waiting to have a policy that’s out, or a legislation that’s out, and then actually have very little ways of enforcing it. [7]*

*It needs to be co-ordinated. It doesn’t really matter who is leading, as long as it is co-ordinated… [2]*

#### IIIc2. Regulatory structure: ‘It has to have some bite’

Experts concluded that enforcement needs to be strict, with defined and material consequences. Enforcement also requires a pre-planned monitoring strategy with a specified budget. However, experts argued that current regulations lack consequences and that regulatory and consumer agencies are, in their view, all underfunded.

*I don’t know what the right scenario would be. We have got to have some teeth, because there are no teeth on any of the regulations at the moment. The teeth that really hurts is the restriction from advertising, and public exposure for being an irresponsible member of our society. [1]*

*The place where it hurts these guys most is on corporation tax and VAT, because that goes straight to their bottom line… [2]*

*And yes, like I say, on every subject you need strict enforcement. It can’t be self-regulation, it can’t be just like naming and shaming, you need to actually have significant fines for when companies are in breach of it. [3]*

*You really would need to ramp up the funding of these enforcement agencies. We know they’re already often sadly underfunded, consumer authorities, and at least ideally this wouldn’t just be a policy in only the UK and in that case you would need a lot of cross-border cooperation. [3]*

*Having a plan for maintaining and monitoring and enforcement … [and] a budget for that being very strong in the beginning and less strong later. [4]*

Civil society actors should also be able to bring challenges to the regulation and its implementation. Experts gave the cautionary example of the UK AADC that relies on the UK’s Information Commissioner’s Office to implement and does not permit individuals to make complaints.

*What’s needed in any of these is then routes for individuals and groups, and civil society organisations to then take action on behalf of individuals. So I think similarly to this and similarly to the ad stuff, the harm isn’t so, it’s not like being physically harmed in the immediacy, there’s a kind of build-up of the effects of it, as well as obviously sometimes individual pieces of advertising have particularly harmful effects, and that applies to both areas that we work in. [7]*

### IV. The big picture

Across all the interviews, experts stressed the importance of understanding and addressing HFSS online marketing as part of the multiple systems in which it is embedded. These considerations included the regulation’s fit with different advocacy agendas, how it interacts with other types of policies and the broader impact of digital marketing on social norms.

#### IVa. Advocacy agenda: ‘Coherence of rules is important’

As online HFSS marketing is embedded within media, digital and food systems, interviewees viewed it differently depending on their expertise, though all felt that addressing digital marketing as part of an overall system would facilitate implementation by platforms, as regulatory requirements would be more consistent and coherent.

Experts with a background in regulating more traditional forms of unhealthy food marketing to children (e.g. TV, product packaging) saw the logical home of this regulation within unhealthy food marketing more generally. They believed that all HFSS marketing restrictions (including digital marketing) should be consistent and comprehensive.

*I think that it must come with other restrictions, so, not just Internet, so also restrictions in marketing in TV (even if it’s decreasing, it’s important, at least here [Chile], radio, billboards), I mean, what we have seen is that the coherence of rules is important, … they cannot have a price promotion either, … you cannot have advice to eat something in some measures, and to restrict the buying this particular or purchasing this particular food item. … coherent steps I think we have learned that [4]*

Experts whose background was in digital media systems, advertising, creative media and/or child rights believed that HFSS digital policy should be considered within the full range of potential online harms.

*
Banning digital marketing of food – can you just tell me why we would do that and then leave self-harm or disinformation or cosmetic surgery or…? … This is my question: why is this sitting somewhere else? Why is it sitting outside of the bigger question about how we want to live our lives, what’s acceptable on and offline and what the food companies should be doing, what advertising should be doing? I just don’t understand why it sits there on its own in that way…… solutions have to sit within bigger understandings. [8]*

*[as an online harm] - that is where the skills will be, and that is where the right kind of legislation will be. [1]*

*It is not just specific to alcohol or to HFSS or to misinformation or to counterterrorism or to sexual abuse online. The principles are all equally applicable, that there are certain levels of content that shouldn’t be shown to people, and there has to be appropriate processes and controls in place to make sure that the platforms are being used responsibly by users and by advertisers. [2]*

Experts in a variety of domains also proposed that HFSS online marketing belonged within the practice of surveillance advertising, where data from internet users (including children) are extracted to build profiles of them and their characteristics and interests to employ for targeting advertising.

*And just for the record, I think all targeted advertising to children should be banned right now, period, end of. [8]*

*It is incredible that we’re all comfortable with companies having so much data about children. [5]*

*That you cannot use personal data in the provisions of advertising. [7]*

*People don’t really argue much about the need to protect privacy, and so combining this data protection, privacy, autonomy and public health ethos together as a package might work to make a compelling interest. [6]*

*I would ban surveillance-based advertising; I think it’s the thing that springs to mind because that would get rid of a lot of influence earned ads outside of the content marketing. And then of course, you’re also left with huge problems with content marketing, but I think that needs more tools and honestly, I don’t have the solution to that. So I think if you start by removing a lot of this targeted advertising then you also remove a lot of access to data which is used in other advertising as well. [3]*

An advantage to focusing on digital marketing as surveillance marketing is that these concerns are cross-regional and include cross-party allegiances in the UK, Europe and the USA.

*There’s a movement now, both sides of the Atlantic, to ban all digital advertising, data-driven advertising, in the UK and EU and US, and all data-driven advertising would be prohibited, so clearly food and beverage marketing would be a part of it. That’s beyond what you guys are looking at but it’s something to think about. [6]*

*I think these kind of issues percolate really nicely in the middle, and indeed a lot of the [UK] support that we have got for the children and ills of targeted advertising has been from Conservative politicians, and we got much more interest, for instance, from the right wing press than from the left wing press… the EU is currently considering policy around this, around regulating digital marketing generally. … and this has got support from the Greens and the very Lefty parties all the way through to, they’ve got one or two people from the centre-right, which is really important, we need to break down that barrier of this being a left issue and a right issue. [in the US]… both a Republican and a Democrat … proposing the legislation [7]*

#### IVb. Interaction with other policies: ‘An opportunity on the European level’

Experts saw an opportunity to focus on the global need to end surveillance and other manipulative forms of marketing online, as exemplified by the forthcoming EU’s Digital Services Act in particular. As noted earlier, several also identified synergies with the issues related to online harms more generally.

*They are debating the Digital Services Act … amendments for that that would ban surveillance advertising in the whole. There is also similar initiatives going on in the US to advocate for banning targeted advertising, and there are two kind of bills going through the US system at the moment. [7]*

*So, I think there’s an opportunity on the European level you have the Digital Services Act coming up, where we talked about platform responsibility and actually being able to hold platforms responsible for paid content such as advertising, I think could help because that would actually put some burden on the platforms to separate paid content and user-created content that’s not promoted. [3]*

However, some cautioned against the issue of creating strong laws and regulations that then fail at the implementation and enforcement stage, such as the GDPR and potentially the AADC.

*Both of those [AADC and GDPR], which are the two pieces that we really have in the UK, both of those certainly have their challenges. What I struggle with is that in the UK we didn’t even do some of those really basic [GDPR] enforcement, and yet have implemented this hugely ambitious [AADC] going way beyond what anybody else is doing anywhere in the world as far as I can see. [7]*

#### IVc. Social norms: ‘We are slowly moving there’

Finally, and returning to the longer-term impact of regulation, the experts from Chile pointed to the potential that even imperfect regulation could start the process of changing social norms. They described children pointing out the ‘High In’ logo used in Chile to identify HFSS foods. They predicted that as resistance to HFSS foods begins to come from children and families themselves, in response to government action, it then drives further change by stakeholders.

*Going to the supermarket … you could see … parents bargaining with … their small young kids like ‘OK but it has just one logo’. And of course if you can get to these young kids and then, of course we know they [influence] what a family will buy and - that is just for small kids not for adolescents but then they are growing up with these new ideas … environment is very important for young kids … it’s an additive effect of these things, when you start understanding that they are no longer desirable things to do. [4]*

*Social norms are also a thing that it’s important to consider. At the end it cannot be any monitoring or enforcement system but the thing that they want - as the social norms change… It’s like being racist now, or discriminating [against] women you won’t do that even if you think that you could - you won’t do that because you will be destroyed by the population. So I think we will get there and we are slowly moving there. [4]*

The South America experts pointed to anecdotal perceptions of changing social norms indicating the potential for changes in relationships within families and with food marketing, advertisers and retailers. They noted how in Chile, where a wide-ranging (but not fully comprehensive) HFSS food marketing regulation was implemented, they had seen changes in perceptions of children, retailers and others regarding what is acceptable. They believed that these changes had implications for social norms of eating and that retailers would need to respond.

## Discussion

Expert insights into the UK’s ‘total ban’ on paid product advertising in digital media, as originally proposed in November 2020^(27)^ for the subsequent Health and Care Act (2022; paused until 2025), highlight a policy that they praised as ground-breaking while, at the same time, also being too limited in scope. Note that the policy they discussed still included ‘owned’ social media advertising in its scope, excluded in the government’s 2021 consultation response^(28)^ and the final 2022 Act.

The policy, experts agreed, establishes a key principle of protecting citizens, particularly children, from the deleterious effects of digital marketing for unhealthy foods. Yet they were of the view that the policy as designed – particularly in its focus on product marketing rather than brand marketing – leaves the door open to industry shifts to forms of impactful marketing that are already well developed. This raises fundamental questions for regulators around the world about how to go about defining such marketing to ensure that regulatory efforts bite. Furthermore, experts insisted that for effective implementation, adequate and creative monitoring and sanctions must be built in.

As noted above, the experts were interviewed before the UK Parliament had passed the design of the Health and Care Act (2022), which is currently scheduled to take effect in 2025; they applauded the UK for being the first country to propose government regulations to limit online HFSS marketing, thus establishing a key principle with the potential to spur other countries to take action, as all such action helps to address health inequities and children’s rights. The importance of a country making the first regulatory move has been demonstrated in other food policy initiatives. Ten years after the first sugar-sweetened beverage tax was implemented in Mexico in 2013, fifty countries had enacted such taxes^(41)^. Similarly, 12 years after Chile enacted some of the strictest front-of-package labelling requirements, thirty countries have proposed similar laws^(42)^. Experts also agreed that the regulation is an important step to help address health disparities affecting individuals of lower socio-economic position. Therefore, any regulation that reduces unhealthy food marketing will disproportionately benefit these individuals. In addition, this legislation would help address common HFSS digital marketing practices that threaten children’s rights to health, privacy and freedom from exploitation. Under the UNCRC, signatories (including the UK) are required to protect these and other rights^(40)^.

The experts also highlighted the ‘all-ages’ approach as a positive feature of the proposed UK legislation. Many felt that the proposed approach, of banning all HFSS product advertising regardless of media audience age profile, is likely to be more effective than existing marketing policies that only limit marketing in media primarily viewed by and/or specifically targeted to children (e.g. children’s programming). The 'all-ages' feature would preclude companies from simply moving their advertising to media widely viewed by adults, which usually includes large numbers of children in the audience too (e.g. in TV, family or sports programming), a common industry tactic to neutralise effects of other marketing regulations. For example, after the UK passed legislation to restrict HFSS television advertising directed to children in 2007, HFSS advertising spots during airtime classed as ‘children’s’ TV were virtually eliminated^(43)^. However, HFSS advertising around programming in family and adult airtime increased. As a result, total exposure to HFSS advertising for all viewers, including children, *increased* following the regulation^(44,45)^. Evaluations of food industry self-regulation in a number of countries have similarly found that even when children’s exposure to unhealthy food advertising during children’s TV programming declined, their exposure to unhealthy food advertising across all types of television programming increased^(10)^.

Experts also commented that the decision to enact government regulation, rather than to rely on industry self-regulation, was crucial for effective policy. Numerous evaluations of food industry self-regulatory policies have demonstrated little to no reductions in children’s exposure to unhealthy food advertising^(10,19,23)^. Moreover, experts discussed unique features of the digital advertising ecosystem that further reduce the likelihood of effective self-regulation. They observed that a few major platforms’ global dominance (e.g. Google, Meta, Amazon) allows them to block outside scrutiny of their practices by restricting access to internal data, while advertisers and platforms both claim they are not responsible for ad delivery. For example, the experts predicted that the effects of Google’s policy to not allow HFSS advertising to accounts self-identified as under 18 in the EU and UK are likely to be limited as few children self-identify as under 18 in Google or YouTube. Yet it is impossible to know without the ability to monitor Googles’ policy from the outside.

One major area that the experts did not discuss was the feasibility of implementing any type of marketing regulations within the current geo-political context. This likely reflects the lack of legal experts among the expert participants included in the study. Powerful transnational corporations have successfully invested enormous resources to effectively neutralise introductions of effective policy actions to address the harms caused by HFSS marketing^(46)^. Accordingly, the industry body for digital advertising (Internet Advertising Bureau (IAB), a global network of forty-five organisations including one based in the UK) has taken credit for the delay of the introduction of the proposed UK policy until at least October 2025, following lobbying by the IAB, its members and their industry partners^(47)^.

### Experts’ recommendations

Despite the many benefits of the proposed policy, experts concluded that the UK restrictions as originally specified in 2020^(27)^ (i.e. restrictions that were stronger than the final ‘ban’ enacted in 2022) had grave limitations as, despite being described as a ‘total’ ban, it would in fact only capture a relatively small proportion of the current online marketing mix. It would not cover most digital marketing that embeds brands (but not specific products) into entertainment content (e.g. in online games, music, sponsorships, programming). The brand activation market (i.e. content marketing other than paid online ads) is estimated to be three times the size of the paid online advertising market currently covered by the regulation^(48,49)^. These and various other forms of branded ‘advertainment’ are rapidly displacing more traditional forms of paid online advertising. In particular, marketing embedded in online games has been described as the ‘wild west’ of the internet with little oversight, and the metaverse could potentially lead to even greater submersion of marketing into entertainment content^(17)^, although some question the long-term potential of the metaverse^(50)^. Moreover, the experts predicted that online marketing will quickly shift to further newer strategies not included in the ban.

Despite their overall support of the original (November 2020) proposed UK legislation to restrict HFSS online marketing, the experts had many recommendations to address these major gaps in its scope. All agreed that brand marketing must be covered – a significant loophole in the legislation. The industry has been able to exploit this loophole in all existing food marketing regulations that only restrict advertising of foods that do not meet nutritional requirements by simply picturing their brand logo (and not a specific food) or by picturing another ‘healthier’ variety (e.g. Coke Zero) instead of their flagship brand (Coke). However, as the marketing experts interviewed explained, all marketing is designed to support a brand (not only the products pictured). Companies will do whatever is necessary to protect their investment in their brands, which can be worth billions (an estimated US$73 billion for Coca-Cola)^(51)^. Targeting children with brand marketing, to firmly establish brand preference, also results in lifelong loyal customers. As a result, much of this brand marketing is designed to appeal to young people by tapping into their core developmental needs for social affiliation and identity development. Food companies boast to shareholders about their efforts to engage with young consumers and embed their brands into youth culture^(17)^.

Advertainment (entertainment created for advertising purposes) is also creating an online promotional world in which brand content, games, apps and other events are activities that consumers actively seek out. In addition, advertainment raises many concerns about its exploitation of young people^(18)^. This type of marketing is more difficult to recognise and actively defend against than traditional paid advertising. Even when recognised as marketing, the entertainment content distracts from and effectively deactivates sceptical responses to marketing. Advertainment is also intended to condition positive attitudes and thus increase consumption by creating positive emotional associations with brands through a classical conditioning process^(52)^. Thus, many consider these forms of ‘stealth’ marketing to be unfair and deceptive, especially when aimed at children.

As noted throughout, a key limitation of the final UK 2022 Health and Care Act is that, although it restricts HFSS marketing in ‘paid’ media, it does not encompass ‘owned’ media as originally proposed in 2020. Notably, ‘earned’ media was not in scope at any stage. ‘Earned’ media is spread virally by online users: common examples include influencers that mention a brand in their videos (without necessarily being paid directly by the brand, but they may have been ‘gifted’ products) and user-generated social media posts. Youth exposure to these messages can be substantial. For example, almost one-half of videos on the most popular YouTube child influencer channels contained branded food appearances^(53)^. Companies generate earned media through marketing campaigns designed to generate ‘buzz’, but they are not directly responsible for its content or dissemination. In addition, the policy restrictions may not address future developments in the rapidly changing world of online marketing, including potential adoptions of virtual reality and/or artificial intelligence for marketing purposes. It will be imperative to define the principles behind online marketing regulations (e.g. companies should not market harmful products, target children and/or engage in surveillance marketing), rather than attempt to focus on specific marketing techniques covered, so the policy does not become outdated as soon as it is launched.

The experts also re-iterated unique technical challenges to regulating digital marketing that have been well-documented^(9,16,33–35)^. Complexities of the digital marketing ecosystem make it extremely difficult to monitor and enforce compliance with regulation. Interestingly, experts expressed differing opinions about whether the large digital platforms and advertisers even know who is viewing online ads. Many commented that effective implementation of digital regulations would require cooperation of the large digital platforms (Google, Meta, Amazon), as advertising served within the ‘black boxes’ of these platforms and other sites requiring sign-in is particularly challenging to assess from outside. Experts recommended that these platforms be required to share this information. For example, they could be required to create complete and transparent ‘Ad Libraries’ for monitoring and analysis, which would also provide outside access to marketing that currently cannot be seen by online ‘bots’ or web crawlers. Another option would be for regulations to require companies to opt-in to online advertising by demonstrating that their food products and advertising strategies all meet defined nutrition thresholds and agreed formats before they could be marketed online.

Nonetheless, the expert view was that industry players currently have little incentive to cooperate with outside regulators. Thus, effective digital marketing regulations also require strong enforcement mechanisms. Experts believed that if regulatory oversight ‘has teeth’, then platforms will develop the technical capacity to comply, as they have found a way to do with copyrighted content. To do so, monitoring must be proactive and adequately funded and non-compliance must result in substantial penalties. In addition, multiple agencies with effective co-ordination must be involved in HFSS regulation, including those covering online, media, food and competition domains. Regulators could also establish a mechanism for civil society to identify and report breaches, rather than relying entirely on the regulator. According to the experts, early experience from Chile suggests that social norms and business practices may begin to shift when strong rules are created and enforced.

Finally, a number of the experts’ comments and suggestions for alternative approaches can help inform future regulations. For example, in addition to banning HFSS online food marketing, such laws could restrict marketing of other harmful products and online practices that threaten children’s health (e.g. tobacco, alcohol, gambling). This expanded focus on broader ‘harms’ could increase support by enlisting the cooperation and efforts of other advocacy groups. In addition to enhancing feasibility and effectiveness, the UK policy approach to restrict HFSS online marketing to all age groups bypasses the need to establish an outside mechanism to identify and verify individuals’ age (such as registration process), thus avoiding numerous issues about privacy and surveillance by the experts interviewed. Another potential approach for policies designed to restrict online marketing to children would be to require adults to opt into receiving HFSS marketing online, which would help address both privacy and feasibility issues.

As targeted digital food marketing, which often utilises data based on interests, location and other personal factors to deliver advertising content, is also a threat to privacy (in addition to or in place of the harmful quality of the products marketed), many experts considered a ban on such ‘surveillance’ marketing to be the most promising regulatory approach, as they believed this would address broader concerns about online privacy that would garner broader support across countries and regions and fit with policies already enacted or under consideration (e.g. COPPA, GDPR). Many of these concerns now also revolve around the growth of ‘artificial intelligence’ data analysis techniques via machine learning, which amplify marketing effectiveness, including but not limited to location-based and interest-based targeting based on personal data extraction, which clearly threaten children’s rights to privacy and freedom from exploitation under the UNCRC^(39)^.

### Strengths and limitations

As with all qualitative research, these findings reflect the areas of expertise and biases of the experts interviewed. The aim of the study, to interview national regional and global experts in this complex interdisciplinary domain, necessarily limited the number of potential participants, but we were able to include experts in all relevant disciplines, except the law. Two had deep experience of the digital infrastructure and marketing industries, and all were involved, to varying degrees, with public health advocacy for marketing and/or online government policies and were thus interested in regulatory solutions. This means that the findings as presented here reflect a public-health orientation, but this could also be considered a strength as the industry position on these issues has been well documented^(47)^. Additionally, in highly specialised yet interdisciplinary fields, where specific complex expertise is scarce, including more experts does not necessarily lead to deeper insights. Furthermore, although expert views have been sought on the wider UK government obesity policy strategy proposed in July 2020^(54)^ this is the first exploration of expert perspectives of which we are aware that specifically focuses on the *digital* marketing restriction proposal of November 2020^(27)^ and benefits from diverse socio-technical public health expertise.

The findings reflect the specific questions asked in the interviews, although all experts were asked to comment on additional topics that they felt were relevant and important. To reduce researcher bias while still achieving consensus, four researchers, including one who was not involved in the original research design and data collection, participated in the thematic analysis, as recommended by the iterative CQR approach to avoid groupthink within the research team. However, the thematic analysis likely reflects the interests of the researchers, whose research and policy expertise focuses on public health, food marketing and children’s well-being. Finally, we note that since the interviews were conducted, the UK government’s 2021 consultation response removed ‘owned’ media from the proposed restrictions, thus reducing the scope of the Health and Care Act 2022. The Act was passed but the UK government delayed its introduction to 2025.

### Conclusion

Public health advocates and others including policymakers concerned about children increasingly recognise that marketing is a powerful channel, a key commercial determinant of health that fuels corporate profits at the expense of children and society^(46)^. Moreover, the UK and other governments are mandated to act to protect children’s rights under the UNCRC. Any policy will have strengths and weaknesses, but a failure to act at all to address the harmful impact of HFSS online marketing would be to ignore States’ duties under the UNCRC. Online marketing threatens children’s rights to health, good nutrition, privacy and freedom from economic exploitation, and experts predicted these threats will increase with the continued advent of new, more sophisticated and powerful forms of online marketing. Thus, the UK HFSS online food advertising restriction, even with the substantial limitations identified by experts in the original version proposed in November 2020, and allowing for the added weakness of the removal of ‘owned’ media in the final Health and Care Act 2022, still represents an important first step in articulating the principle of curbing predatory industry practices and reducing their impact on children’s health and well-being.

## Supporting information

Harris et al. supplementary material Harris et al. supplementary material

## Supplementary material

For supplementary material referred to in this article, please visit https://doi.org/10.1017/S0007114523002829
