# Supplementary material for: A ‘major breakthrough’, yet potentially ‘entirely ineffective’? Experts’ opinions about the ‘total ban’ on unhealthy food marketing online in the UK’s Health and Care Act (2022)
Source: Br J Nutr. 2025 Mar 28;133(6):763–80. doi: 10.1017/S0007114523002829 (PMC12169946; doi:10.1017/S0007114523002829)
Supplement: Harris et al. supplementary material [file S0007114523002829sup001.docx]

**Semi-structured interview topic guide**

**Introductions**

Thanks so much for taking the time to speak with me today!

*[Interviewer introduces self]*

As you know we are interested in the regulation of digital marketing of food and non-alcoholic beverages and, given your expertise, we’re really keen to hear your thoughts on this.

Specifically, we want to understand your views on *policies to regulate food marketing (not necessarily focused on children only). What should these be? How can they be designed and implemented?*

We’re interested in all angles: political, legal, technical and practical, commercial ….

This study is addressing UK policy but we are keen to explore the global context – we’d like to learn from policy discussions, development and potential implementation in other countries too.

Just before we get going - a reminder that we are recording our conversation, and this will be password protected and later transcribed.

To double check - as you are an expert in your field, we’re happy to use your identity for this piece of research and any resulting publications (and attribute quotes to you by name). But if you prefer to remain anonymous that is also absolutely fine. You’ve said on your consent form that [*cite preference, role as described on form*] – can I just double check that this is still the case?

As you probably know, the UK is planning a ban on digital marketing of unhealthy foods and drinks. *Interviewer to give brief overview of current proposal or further details if interviewee less familiar.*

**Overview question**

I have a set of detailed questions here, but perhaps its most helpful to take an overall view first:

Regarding policies to regulate food marketing, in your view (*please consider an ideal world, as well as what is practical/likely to be acceptable politically etc*),

- What should these be?
- How can these policies be designed and implemented?
- Are you aware of any examples of effective digital marketing regulation?
- How can we ensure it doesn’t exacerbate inequalities (*applies to all questions*)?

**Core questions**

QUESTIONS to be tailored according to the expertise of the participant.

*Note that focus of questions is dependent upon participant time available and their expertise. Therefore*

- *Marketers and marketing specialists will be asked more about marketing design and strategy.*
- *Technical experts will be asked more about technical aspects of digital marketing delivery and monitoring.*
- *Policy experts will be asked more about international regulation and policy.*
- *Everyone was asked about futureproofing.*

Defining scope and criteria

*How do we ensure a digital marketing policy is sufficiently comprehensive/proportionate?*

*What should be in scope?*

Prompts:

- Digital platforms/settings
- Marketing techniques
  - Exposure (reach and frequency),
  - Power (creative content, persuasive techniques)
  - Paid media only? Owned? Earned? Influencers
  - Engagement prompts – likes, shares?
  - Ad format (e.g*., radio, audio only ads, video on demand ads, OOH and delivery ads, advergames*, *display, desktop and mobile video, digital out of home, connected TV, paid search, branded content/publisher partnerships, content-recommendation/discovery, in-game, paid social media, user generated content, owned website/blog, search engine optimisation, email, organic social) –* what to consider for those that are outside of practical control (e.g., scheduling)?
  - Do you think it would be proportional to include SMEs in advertising bans?
- Marketing currently out of scope
  - Brand marketing, CSR, sponsorship, delivery, should these be incorporated? if so, how?

Monitoring and enforcement

*How can these regulations be implemented effectively?*

*Given the highly distributed and personalised nature of advertising delivery in the digital economy, can it be monitored?*

- Are there best methods?
- Nordic joint monitoring protocol – has it been effective, can we learn from this?
- Mechanisms could be pre-vetting (proactive) or complaint based (reactive) – what are implications?
- Who should have oversight and enforcement responsibility?
- What should sanctions be for non-compliance?
- What transparency and accountability requirements should there be?

Futureproofing

*Can we capture potential future developments in current policy?*

- What are the current trends in digital marketing?
  - strategies, techniques, structural features
  - shift to more contextual marketing?
  - what may change in the next 5-10 years?
  - How might we plan for this with the current regulation, or what additional regulation might be needed in the future to combat this?
  - If  the total restriction of online advertising of HFSS products is implemented as consulted on, where will advertisers go next? What are the key areas to focus on?
- Are there any specific lessons to be learnt from alcohol/tobacco controls?

Existing policies

*About media communications/health policies that include (explicitly or otherwise) online media (e.g., national legislation or voluntary codes in Austria, Germany, Slovenia, Finland, Ireland, the Quebec Consumer Protection Act):*

- Are you aware of any country/ies with regulations that address digital marketing?
- Are you aware of any country currently considering introducing digital food marketing controls? What specifically is being considered?
- Are you aware of countries where a proposed digital marketing regulation was challenged?
  - If yes, by whom? How? and what was the outcome?
- Do you have experience of a digital food marketing policy cycle?
  - if the policy was not implemented, what were the main barriers? What lessons can be learnt?

Relative roles and responsibilities: National Governments / global platforms / marketers

*Who has power to regulate and control over restricting marketing?*

- Is this sort of regulation possible at the national level?
  - Does it require coordinated multi-country action and if so, how might that be achieved? national domestic regulation with supra-national regulations?
- Cross-border communications issues
  - global cross-border dataflows, different concepts of privacy and data protection between jurisdictions, how to fit regulation within this system.

Unintended consequences and economic arguments regulations Economic implications of digital food marketing policies

- Exacerbating inequalities

The interaction between digital marketing regulation and UK Age-Appropriate Design Code (AADC)

- How does the AADC align with regulation of marketing?
- Can a Rights framework be used for digital food marketing policy development? If so, how does it help?

The EU AudioVisual Media Services directive, GDPR

*Do these have a role in underpinning digital marketing regulation across the EU?*

- Strengths/weakness of AVMSD for tackling this issue.
- Strengths/weakness of GDPR for tackling this issue
- Should the UK approach integrate AVMSD principles into any proposed legislation?
- EU DSA and AI Proposal – do you see any potential for digital marketing regulation there?

Consistency with broadcast regulations

*Can digital marketing policies be consistent with current broadcast policies?*

- Where do key differences lie?
- Is broadcast ending anyway? (personalised advertising)
- Watershed v total ban – practicality and efficacy
- Exemptions process? Suggested nature of this?

**Wrapping up**

- What are the main loopholes to look out for? How might we close them?
- What are key challenges for the successful design and implementation of an effective digital food marketing policy? *(Political, legal, technical, practical)*

**Supplemental Table 1: Expert interviewees contributing to the study***

| **Interviewee (expertise, country/region)** | **Current role** | **Expertise and relevant professional history** |
| --- | --- | --- |
| 1  Marketing industry, UK | Creative digital, social, and TV campaigns to encourage children’s healthy eating. Senior UK Government policy adviser | Extensive digital marketing, advertising, app development, image recognition and location targeting expertise. |
| 2  Digital and marketing industry, UK and global | Global advisor, mobile data & advertising, NGOs, industry bodies and UK government. | Digital advertising and technology. Mobile first strategies for major platforms. |
| 3  Digital privacy protection, Europe | Privacy-focused NGO – national and EU-wide. | New technology and consumer protection regarding data, privacy, algorithmic decision making and profiling, policy. |
| 4  Marketing and children/obesity, South America | University Professor | Childhood obesity (basic research, clinical research, applications to public health, particularly food marketing including labelling), children and food marketing, nutrition and policy. |
| 5  Marketing and children/obesity, North and South America | University Researcher | Governmental regulation and policy, children and food marketing. |
| 6  Marketing and children/obesity, digital privacy protection, North America | Executive Director, NGO | Digital industry analyst, policy advocate, and leading global advocate for restricting digital marketing to children. |
| 7  Marketing and privacy policy, UK | Senior Researcher, UK NGO | NGO research and advocacy, specialises in formulating policy proposals. |
| 8  Children’s digital and privacy protection and rights, UK and global | Founding Chair of a global foundation; UK Parliament. | Child rights, digital media, policy development. |

**Participants agreed to be quoted in reporting of the research.*

**Proposed design of the UK online HFSS advertising restrictions (as was available at the time of the interviews)**

**Advertising in scope**

We propose that the restrictions apply to all online marketing communications that are either intended or likely to come to the attention of UK consumers and which have the effect of promoting identifiable HFSS products, while excluding from scope:

- marketing communications in online media targeted exclusively at business-to-business. We do not seek to limit advertisers' capacity to promote their products and services to other companies or other operators in the supply chain
- factual claims about products and services
- communications with the principal purpose of facilitating an online sale

The scope of the restriction would include, but is not limited to, for example:

- commercial email, commercial text messaging and other messaging services
- marketers' activities in non-paid for space, for example on their website and on social media, where the marketer has editorial and/or financial control over the content
- online display ads in paid-for space (including banner ads and pre/mid-roll video ads)
- paid-for search listings; preferential listings on price comparison sites
- viral advertisements (where content is considered to have been created by the marketer or a third party paid by the marketer or acting under the editorial control of the marketer, with the specific intention of being widely shared. Not content solely on the grounds it has gone viral)
- paid-for advertisements on social media channels - native content, influencers etc
- in-game advertisements
- commercial classified advertisements
- advertisements which are pushed electronically to devices
- advertisements distributed through web widgets
- in-app advertising or apps intended to advertise
- advergames
- advertorials

**Factual claims**

We recognise that companies should be able to make available factual information about their products. Therefore we propose that advertisers remain able to feature such information on their own websites or other non-paid-for space online under their control, including their own social media channels.

We consider that factual claims include but are not limited to:

- the names of products
- nutritional information
- price statements
- product ingredients
- name and contact details of the advertiser
- provenance of ingredients
- health warnings and serving recommendations
- availability or location of products
- corporate information on, for example, the sales performance of a product

However, we note in this context the regulatory challenges arising from having to make a distinction between factual claims and promotional claims, and the inherently shareable and engaging nature of social media content. This is highlighted by recent partially upheld [ASA rulings](https://www.asa.org.uk/codes-and-rulings/rulings.html?q=&sort_order=relevant&custom_date=1&from_date=18%2F12%2F2019&to_date=18%2F12%2F2019&topic=83B823B2-DEBA-498A-AA709E19387E58A4) against 4 e-cigarette advertisers in December 2019 which concluded in all cases that the advertisers should take steps to ensure that claims made on their social media channels should only be distributed to those actively following those channels and should not be seen by other users.

We therefore propose that any advertisers which sell or promote an identifiable HFSS product or which operate a brand considered by the regulator to be synonymous with HFSS products should be required to set controls which ensure that their posts regarding HFSS products can only be found by users actively seeking them on the advertisers own social media page. This could be achieved, for example, by ensuring that the privacy settings on their social media channels are set so that their content appears on that page only.

**Online sales**

We also want to ensure that any advertiser who uses the internet to conduct transactions of their products is allowed to continue selling their products online. We therefore propose any platform whose principal function is the buying or selling of products, including food and drink, is exempt from the proposed restriction. This includes websites, social media channels, apps ‒ or dissociable part of those platforms, including also email, text or push notifications directed to customers who have chosen to opt-in to these communications.

Audience measurement and the treatment of BVoD platforms

As noted above one of the key drivers for government proposing a total online restriction is the absence of any independent, comprehensive, gold-standard and publicly available means of audience measurement online. This is in contrast to TV, where Broadcasters Audience Research Board (BARB) data provides a level of assurance to advertisers which is not widely available online. A broadcaster-led initiative to deliver multiple-screen programme viewing figures (Project Dovetail) now means that broadcast video on demand (BVoD) platforms can depend on the same standard of audience measurement as linear broadcast. We propose applying a watershed to the adverts shown instream during programming on BVoD platforms to mirror our approach to linear TV, separate to the approach for other online media.

**Liability**

Here we seek to build on existing regulatory structures in order to minimise disruption to industry and regulators. We also want to ensure that online advertising regulation sufficiently incentivises compliance and drives rapid remedial action.

We will appoint a statutory regulator with overall responsibility for the regulation of the restriction, with discretionary powers to take effective action against advertisers who breach the rules, especially in cases of more serious or repeat breaches. We propose that the day-to-day responsibility for applying the rules, considering complaints, provisioning guidance and training material to industry would remain with the ASA, recognising their expertise and experience in regulating advertising.

We propose that advertisers are liable for compliance with a total online HFSS advertising restriction.

In addition we want to consider whether other actors in the online advertising ecosystem should have responsibility for advertising that breaches an online restriction. We envisage that the nature of this responsibility would depend on the level of control which the actor had over the advertising that was served on their sites or placed through their ad networks.

We consider that there is scope to introduce such requirements through specific legislation that prohibits these actors from running advertising that breaches the restriction.

We also recognise that there is scope for legislation to set out a requirement to introduce measures appropriate to the level of control that the actor has over advertising in order to prevent the dissemination of advertising in breach of the restriction. The regulator would then be responsible for ensuring compliance with and enforcement of such appropriate measures. Guidance on appropriate measures would be developed in consultation with industry and other stakeholders in an open and transparent way, with the regulator ultimately responsible for determining the content.

We will consider the use of a requirement for the takedown of advertising that breaches the restriction after it has been brought to the relevant ad networks' attention. This would not affect protections under the UK's intermediary liability regime which limits liability for illegal third party content hosted on online services until the service provider has received notification of its existence and they have subsequently failed to remove it from their services in good time.

**Enforcement**

We want to ensure that the enforcement powers of the statutory regulator are designed and used in a way that incentivises compliance and allows for rapid remedial action.

We propose that the day-to-day responsibility for applying the restriction, considering complaints about advertising that breaches the restriction, provisioning guidance and training material to the advertising industry would be given to the ASA, recognising their expertise and experience in regulating advertising.

In line with the current regulatory regime, we propose that breaches would be resolved in line with current ASA policy of responding to individual complaints and promoting voluntary cooperation with the restriction. If this approach failed or advertisers were committing repeated or severe breaches relating to HFSS marketing material, they would face stronger penalties through the statutory backstop. We would envisage that these would include civil sanctions, including the ability to issue fines in defined circumstances and tied to defined metrics.

To support any potential requirements on in scope online service providers, we will consider whether it is necessary for the statutory regulator to have powers in relation to the oversight of any appropriate measures. These powers might include (but are not limited to):

- requiring evidence of effective maintenance, review and enforcement of the service provider's appropriate measures, which should reflect guidance issued by the regulator in its codes of practice
- requiring evidence of the number of adverts that are being placed in breach of the restriction, who those advertisers are and action taken to prevent adverts that breach the restriction being placed
- requiring evidence of the processes that the service provider has in place for reporting content in breach of the restriction, the number of reports received and how many of those reports led to action

We envisage that the statutory regulator would not be expected to respond to individual complaints about failures to implement appropriate measures, but would have a role more focused on monitoring and review to ensure that appropriate measures are in place. They would be able to work with the ASA to identify areas or online service providers that require intervention. The regulators would also have a discretionary power to impose civil fines for breaches.

The imposition of civil fines by the statutory regulator would open to challenge through normal court procedures.

We note also in this context, the challenges of applying statutory regulation to persons overseas. It is our intention to restrict the HFSS adverts seen by children in the UK. Given the global nature of online media platforms and advertisers, we welcome views on the extent to which an online total restriction on HFSS advertising in the UK could be made to apply to online advertising served in the UK, but originating from advertisers or intermediaries based overseas. We would also be interested to hear views on whether this restriction may disproportionately affect UK-based companies.

**Additional considerations**

*Public sector equality duty*

As part of the consultation, we are inviting views on the impact of these advertising restrictions on people with protected characteristics and steps that could be taken to mitigate the impact, against the government's duties under the Equality Act 2010.

*Socioeconomic considerations*

In addition to the protected characteristics, we also want to consider the potential for these advertising restrictions to reduce inequality in health outcomes experienced by different socioeconomic groups.
